# Heat-killed *Mycobacterium tuberculosis*-induced trained immunity elicits antitumor activity in PBMCs from colon cancer patients with unresponsiveness to anti-PD-1 *in vitro*

**DOI:** 10.3389/fimmu.2026.1911032

**Published:** 2026-09-11

**Authors:** Gülce Bıçakcıoğlu, Laura Bravo-Robles, Pablo Mata-Martínez, Jaime Fernández, Verónica Terrón-Arcos, Natalia González, Inés Rubio, Rebeca Abad-Moret, Sylwia Bilas, Esteban Díaz, Ramón Cantero-Cid, Eduardo López-Collazo, Carlos del Fresno

**Affiliations:** 1 The Innate Immune Response Group, IdiPAZ, La Paz University Hospital, Madrid, Spain; 2 Immunomodulation Laboratory, IdiPAZ, La Paz University Hospital, Madrid, Spain; 3 Inmunotek, S.L., Alcalá de Henares, Madrid, Spain; 4 Tumorimmunology Laboratory, IdiPAZ, La Paz University Hospital, Madrid, Spain; 5 General and Digestive Surgery Service, La Paz University Hospital, Madrid, Spain; 6 Digestive Surgery Department, Faculty of Medicine, Universidad Autónoma de Madrid, Madrid, Spain; 7 UNIE University, Madrid, Spain; 8 CIBER of Respiratory Diseases (CIBERES), Madrid, Spain; 9 Biobank, IdiPAZ, La Paz University Hospital, Madrid, Spain; 10 CIBER of Cardiovascular Diseases (CIBERCV), Madrid, Spain

**Keywords:** anti-PD-1, colon cancer, HK*Mtb*, immunotherapy, trained immunity

## Abstract

**Introduction:**

Trained immunity (TI) is a form of innate immune memory characterized by boosted innate immune responses to secondary heterologous challenges following an initial stimulus. Several inducers, including fungal β-glucan and the Bacillus Calmette–Guérin (BCG) vaccine, induce TI in human peripheral blood mononuclear cells (PBMCs). Although the potential of TI in colon cancer has been explored in murine models, its relevance in patients remains poorly understood.

**Methods:**

Building on our previous demonstration that heat-killed *Mycobacterium tuberculosis* (HK*Mtb*) induces TI, we applied a standardised training protocol to PBMCs from colon cancer patients (CC patients) and healthy volunteers (HV). Cytokine responses to heterologous stimulation were assessed using multiplex assays. To evaluate functional antitumour relevance, we performed *in vitro* tumour-killing assays against the SW480 colon cancer cell line, in the presence or absence of anti-PD-1 treatment, and characterised the associated cytokine milieu.

**Results:**

HK*Mtb*-trained PBMCs from CC patients exhibited a partially preserved TI phenotype against secondary heterologous challenges, with increased TNF and IFN-γ and reduced IL-10, comparable to HV. By contrast, IL-6 and IL-1β production were not enhanced. Notably, HK*Mtb*-induced training significantly increased the tumour-killing capacity of PBMCs from both CC patients and HV. In CC patients, this effect was accompanied by a specific heightened production of IL-12p70 and IP-10. Furthermore, whereas the anti-PD-1 treatment alone failed to induce a significant antitumour response in PBMCs from CC patients compared with HV, HK*Mtb*-induced TI overcame this lack of response. Of note, this was again associated with an enhanced IL-12p70 and IP-10 profile.

**Conclusion:**

These findings provide proof of concept that HK*Mtb*-induced TI enhances the antitumour activity of PBMCs from CC patients that are unresponsive to PD-1 blockade *in vitro*, supporting its potential as a promising immunomodulatory strategy in non-responsive CC patients.

## 1 Introduction

Cancer is one of the deadliest diseases in the world. According to GLOBOCAN, 19.9 million people were diagnosed with cancer in 2022, and 9.7 million people died from the disease. Among various types of cancer, colorectal cancer (CRC) represents a major global health burden, accounting for approximately 10% of all cancer cases and ranking among the most lethal tumour types (1). Disease recurrence occurs in up to 30% of patients, even after surgery and adjuvant chemotherapy (2). Although colon and rectal cancer are often grouped under the umbrella term CRC, because of shared symptoms, risk factors, biological features and screening strategies, important differences exist between them. GLOBOCAN estimates that colon cancer accounts for around two-thirds of CRC, whereas rectal cancer accounts for the remaining third (1). Rectal cancer is also associated with a higher risk of recurrence, particularly in locally advanced disease (3). Indeed, epidemiological, anatomical, molecular and hereditary differences, together with distinct metastatic patterns and multimodal treatment approaches, support the need to consider colon and rectal cancer as separate clinical and biological entities (4).

Immunotherapy has transformed the treatment landscape of several malignancies; however, its efficacy in CRC remains limited for most patients, particularly when compared with outcomes achieved in melanoma and lung cancer (5). Immune checkpoint inhibitors (ICI)-based therapies, including anti-PD-L1/PD-1 antibodies, have shown meaningful clinical benefit in only 4-5% of metastatic CRC cases. This subgroup is characterized by a deficient mismatch repair system and high microsatellite instability (dMMR: MSI-H) (6). Although these patients are expected to benefit from anti-PD-1 therapy, objective response rates do not exceed 40% (7). This response pattern is partly explained by the hypermutated phenotype seen in the dMMR:MSI-H tumours, which generate abundant neoantigens and an inflamed tumour microenvironment, in which immune checkpoint pathways restrain cytotoxic T-cell activity. By contrast, proficient mismatch repair and microsatellite-stable tumours (pMMR/MSS) are typically immune-excluded, rendering ICI-based therapies largely ineffective (8). In this context, complementary immunomodulatory strategies capable of reshaping the immune landscape are urgently needed to overcome resistance to ICI-based therapies in CRC.

In parallel with the need to improve cancer therapy, increasing evidence indicates that a deeper understanding of immune system organization and responsiveness across tumour types may reveal new therapeutic opportunities. Whereas earlier views placed adaptive immunity at the centre of immunological memory, it is now recognised that the innate immune system can also acquire long-lasting functional adaptations that shape subsequent responses. Innate immune cells can undergo functional reprogramming after defined stimuli, resulting in enhanced responsiveness to secondary challenges. This process, described over the past decade, is known as trained immunity (TI) (9). TI refers to an enhanced innate immune response to secondary heterologous stimulation; it results from epigenetic reprogramming induced by a primary stimulus, and represents a *de facto* innate immune memory (10). The concept emerged from observation of non-specific protective effects following vaccination campaigns, particularly those involving the Bacillus Calmette–Guérin (BCG) vaccine (11, 12).

Several TI inducers have been described, with yeast-derived β-glucan and BCG vaccine as the best-studied. These two stimuli have provided proof of concept for the protective potential of TI against diverse heterologous challenges, such as infections (13–15) and cancer (16, 17). Nevertheless, these TI inducers bear significant limitations. BCG is a live-attenuated vaccine with a compromised safety profile associated with risks of systemic infection or excessive inflammatory responses (18, 19), and the particulate nature of β-glucans and their insolubility (20–22) limit their clinical applicability.

These limitations have prompted the development of new TI inducers with greater translational potential. Preclinical approaches have now included HDL-based nanobiologics targeting NOD2 (23, 24), bacteria-derived nanovesicles (25), and β-glucan-coupled nanoparticles (26). Another promising approach involves immunotherapies based on heat-killed bacteria capable of inducing TI (27). In this sense, MV130, a polybacterial immunotherapy against recurrent respiratory infections, is an example of a TI-inducer (28, 29) with an excellent safety profile in patients (30–32). Moreover, in our previous study, we demonstrated that heat-killed *Mycobacterium tuberculosis* (HK*Mtb*) induced TI both in human PBMCs and monocytes *in vitro*, as well as in preclinical mouse models *in vivo* (33).

Given the capacity of TI to remodel innate immune responses, both classical inducers, such as β-glucan, and newer formulations have been tested against cancer. Systemic administration of β-glucan particles or HDL-based nanobiologics conferred protection against melanoma, pancreatic and lung cancer, either alone or in combination with ICI such as anti-PD-L1 (16, 24, 34, 35). Also, BCG conferred protection against melanoma-induced lung experimental metastasis (36). Notably, some infections have also been described as antitumour TI-inducers, such as intranasal Influenza virus (37) and *Escherichia coli*-induced pneumonia (38) against lung cancer, or *Leishmania braziliensis* against non-Hodgkin lymphoma (39). Despite most of these results coming from preclinical mouse models, clinical studies have demonstrated the efficacy of the BCG vaccine against bladder cancer, coupled to a reduced risk of unrelated respiratory infections in patients receiving this therapy (17).

Of note, data on the effectiveness of TI against colon cancer remain scarce and are largely derived from preclinical studies using mouse models engrafted with tumour cell lines, mainly employing β-glucan particles as TI inducers (20, 22, 40) or as a booster of antitumour vaccines (21, 25). While these studies provide important insights, the ability of TI inducers with clinical potential to elicit trained responses in immune cells from CC patients, and their antitumour effects, remains unexplored. To address this gap, we examined the ability of HK*Mtb* to induce TI in PBMCs from CC patients and enhance antitumour activity, comparing this approach with anti–PD-1 therapy and responses observed in healthy volunteers (HV). Collectively, our findings highlight TI as a potential complementary immunomodulatory strategy for CC patients, capable of enhancing tumour cell killing and overcoming current limitations of immunotherapy.

## 2 Materials and methods

### 2.1 Materials

A comprehensive list of all antibodies, equipment, reagents, instruments, commercial assays, and software used in this study is provided in the **Supplementary Material** (**Supplementary Tables 1–S5**).

### 2.2 Methods

#### 2.2.1 Blood samples

Blood samples were sequentially collected from HV older than 55 years from the Blood Donor Service at La Paz University Hospital (Madrid, Spain). Blood samples from CC patients were recruited from the General Surgery and Digestive Service of La Paz University Hospital before surgery and any medication. To minimize the potential effect of previous systemic treatments, patients receiving chemotherapy, radiotherapy, or other prior systemic therapy were excluded. Informed consent was obtained from all the participants. The study cohort consisted of 17 HV and 18 CC patients. Their demographic characteristics, including age and sex distribution, are summarized in **Table 1**. All CC patients were proficient in the mismatch repair system (pMMR) and showed microsatellite stability (MSS). All the experiments were approved by the La Paz University Hospital Ethics Committee (PI-5270).

**Table 1:** Demographic and clinical characteristics of healthy volunteers (HV) and colon cancer (CC) patients.

Parameter	HV	CC
Age (mean ± SD)	59.8 ± 3.4	67.8 ± 13.8
Sex, n (%)		
Male	13 (76.5%)	15 (83.3%)
Female	4 (23.5%)	3 (16.7%)
Tumor Stage, n (%)		
I	–	3 (16.7%)
II	–	8 (44.4%)
III	–	5 (27.8%)
IV	–	2 (11.1%)
MSI Status	–	MLH1-PMS2-MSH2-MHS6 Preserved (100%)
Pathological T Stage, n (%)		
I	–	3 (16.7%)
II	–	1 (5.6%)
III	–	11 (61%)
IV	–	3 (16.7%)

Blood samples from HV were obtained from the Blood Donors Unit, while blood samples from CC patients were collected before surgery and any treatment at the General and Digestive Service of La Paz University Hospital.

#### 2.2.2 Isolation of PBMCs and culture

Fresh blood samples were collected in ethylenediaminetetraacetic acid (EDTA) anticoagulant tubes (Vacutest, Avantor #720-2770). Peripheral blood mononuclear cells (PBMCs) were isolated by gradient centrifugation on Ficoll-Paque (GE Healthcare, 17-1440-03). 6 mL Ficoll-Paque was slowly topped by 18 mL blood sample. The tube was centrifuged at 450 g for 25 minutes by mid-degree acceleration and without brake. After centrifugation, PBMCs were collected from the middle-gradient layer and washed with phosphate buffer saline (PBS, Sigma-Aldrich, P4417-100TAB) by centrifugation for 5 minutes at 450 g. The pellet was resuspended in 1 mL Pharm/Lyse (BD Biosciences, 554723) and incubated for 1 minute to lyse the remaining red blood cells. Then, the cells were washed again with PBS by centrifugation at 450 g for 5 minutes. The supernatant was aspirated, and the cells were resuspended in 1 mL Roswell Park Memorial Institute (RPMI) medium (Thermo Fisher Scientific, 11594506) supplemented with antibiotics (0.1% penicillin, 0.1% streptomycin, Gibco, 15140-148) and 10% fetal bovine serum (FBS) (Thermo Fisher Scientific, 11560636) (Complete RPMI). Resuspended cells were counted with a dilution of 1:20 trypan blue (Gibco, 15250061) in a Neubauer Hemocytometer (EMS, 68052-15).

#### 2.2.3 Trained immunity induction by HK*Mtb*

For the TI protocol, 0.2 x 10^6^ PBMCs in 100 μL complete RPMI medium were cultured in flat-bottom 96-well plates (Avantor, 734-2337), culturing 6 wells (Day 0). Cells were incubated overnight at 37 °C with 5% CO2 in a humidified incubator (Thermo Scientific, 311). On day 1, 3 wells assigned as the control group were topped with 100 μL RPMI, while, for training, PBMCs were treated with HK*Mtb* (Invivogen, tlrl-hkmt) at a final concentration of 10 μg/mL and a final volume of 200 μL. All PBMCs were incubated for 1 day at 37 °C, 5% CO_2_ in a humidified incubator. After the incubation period, all media in each well were carefully removed, and the wells were gently washed twice with 200 μL PBS. After removing the PBS, 200 μL of complete RPMI was added to the wells, and the cells were incubated for 3 days at 37 °C, 5% CO2 in a humidified incubator. Once the incubation period was over (on day 5), the medium was carefully removed from the wells and gently washed twice with 200 μL PBS. Afterwards, adherent PBMCs from both trained and untrained groups were exposed to 10 ng/mL LPS (Sigma-Aldrich, L4391), 10 μg/mL R848 (Invivogen, tlrl-r848), or just complete RPMI (control group) in a final volume of 200 μL. Stimulated adherent PBMCs were incubated for 1 day at 37 °C, 5% CO_2_ in a humidified incubator. On day 6, the medium in each well was collected in a round-bottom 96-well plate (Avantor, 734-2328) and centrifuged at 450 g for 5 minutes. The supernatant was collected, transferred to another round-bottom 96-well plate, and kept at -20 °C until cytokine measurement procedures were performed.

Of note, this protocol was slightly modified from our previously published *in vitro* model of HK*Mtb* training (33), with a 3-day resting period instead of 5 days. This modification was introduced to ensure sufficient numbers of viable adherent PBMCs following HK*Mtb* training for subsequent co-culture with SW480 tumor cells in the *in vitro* killing assays (see below).). Importantly, we confirmed that the trained heterologous response was comparable after 3 or 5 days of resting (**Supplementary Figure 1**), indicating that this modification did not substantially affect the trained phenotype.

#### 2.2.4 Cytokine measurement

Human TNF-alpha DuoSet ELISA (R&D Systems, DY210) and Human IL-6 DuoSet ELISA (R&D Systems, DY206) were used to quantify TNF and IL-6 cytokines. Calculations were performed following the guidelines provided by the manufacturer. The assay was performed with 1:2 diluted supernatant. The absorbance data were collected via an Epoch 2 Microplate Spectrophotometer (BioTech). A standard curve was fitted to the absorbance data obtained from the spectrophotometer to calculate the concentrations of the cytokines.

The rest of the cytokines were quantified in the supernatants by LEGENDplex™ Human Essential Immune Response Panel through the guidelines provided by the manufacturer (BioLegend, 740930) in a BD FACSCalibur flow cytometer (BD Biosciences, San Jose, CA, USA).

#### 2.2.5 Maintenance of SW480 tumour cells

SW480 colon cancer cell line was purchased from ATCC. SW480 cells were cultured in T75 flasks in 15 mL RPMI medium (Thermo Fisher Scientific, Cat# 11594506) supplemented with antibiotics (0.1% penicillin, 0.1% streptomycin, Gibco, #15140-148), 10% fetal bovine serum (FBS) (Thermo Fisher Scientific, # 11560636), 5% Sodium Pyruvate 100mM (Thermo Fisher Scientific, # 11360070), and 5% GlutaMAXTM (Thermo Fisher Scientific # 35050061).

#### 2.2.6 *In vitro* killing assay

3 x 10^6^ PBMCs previously collected from fresh blood samples were cultured in a 6-well culture plate in 1500 μL complete RPMI on day 0. 2 wells were assigned for each condition (untrained, HK*Mtb* training). Cells were incubated overnight at 37 °C with 5% CO2 in a humidified incubator. On day 1, the wells of the untrained group were topped with complete RPMI, while the training was performed with 10 μg/mL HK*Mtb* in a 3 mL final volume. The cells were incubated overnight at 37 °C with 5% CO2 in a humidified incubator.

On day 2, the media in each well was collected and transferred into a 10 mL tube. Adherent cells were washed with 1 mL PBS, twice, while the tube containing the media was filled with PBS and centrifuged at 450 g for 5 minutes to perform a wash step for the non-adherent cells. The pellet was resuspended in 3 mL complete RPMI and transferred back to the initial wells of each condition. Cells were incubated for 3 days at 37 °C, 5% CO2 in a humidified incubator.

After the incubation period, the media were transferred into a new 25 mL tube from each well. Then, the remaining adherent cells were gently washed twice with 1 mL PBS. To detach them, 1 mL TrypLE™ Express Enzyme (1X) (No Phenol Red - Gibco™, 12604021) was added to the cells and incubated for 7 minutes at 37 °C. After the incubation, detached cells were transferred into the tubes containing the supernatant collected before. All tubes were filled with PBS and centrifuged for 5 minutes at 450 g. The pellet was resuspended in 500 μL complete RPMI and counted with a dilution of 1:5 trypan blue in a Neubauer Hemocytometer.

To perform the assay, SW480 cells were detached from the T75 flask (Sigma-Aldrich, CLS430641U) by using 5 mL of trypsin-EDTA. After detaching the cells, trypsin activity was stopped with 5 mL of RPMI + 10% FBS medium, and the collected cells were washed with PBS in a centrifuge for 5 minutes at 450 g. The pellet containing the cells was resuspended in 1 mL of PBS, and the cells were counted with 1:20 dilution of trypan blue in a Neubauer Hemocytometer. Further, 1 x 10^6^ SW480 were resuspended in 1 mL PBS and stained with CFSE (1 μM) for 20 minutes at 37 °C. Then, 10 mL PBS was added on top, and the cells were centrifuged for 5 minutes at 450 g.

*In vitro* killing assay was designed as a co-culture with a proportion of 1:4 colon cancer cells (SW480) to PBMCs. Hence, 0.03 x 10^6^ SW480 were co-cultured with 0.12 x 10^6^ PBMCs in each well of a flat-bottom 96-well plate to a final volume of 200 μL. For each condition (untrained, trained), 3 wells were prepared.

For the experiments including anti-PD-1 treatment, 3 extra wells from each condition (untrained, trained) were treated with 10 μL of 5 μg/mL anti-PD-1 (Nivolumab). The anti-PD-1 immunotherapy (Nivolumab) was obtained from the Oncology Service of La Paz University Hospital.

After the co-culture and treatment, the *in vitro* killing assay plate was incubated for 48 hours at 37 °C, 5% CO2 in a humidified incubator.

#### 2.2.7 *In vitro* killing assay staining

On Day 7, the killing assay was revealed, and the cells were stained for FACS analysis. The killing assay plate was centrifuged at 90 g for 3 minutes, and the supernatant was kept at – 80 °C for further analyses. All of the wells were washed gently twice with 100 μL. After the last washing, PBS was discarded, and 100 μL trypsin-EDTA was added to each well. The plate was incubated for 10 minutes at 37 °C. After the incubation, all the cells were detached and transferred into a round-bottom 96-well plate. Trypsin was stopped with 100 μL of complete RPMI, and the plate was centrifuged for 5 minutes at 400 g. After discarding the supernatant, wells with the same experimental condition were joined in 200 μL PBS and centrifuged again for 5 minutes at 450 g.

The supernatant was discarded, and all the cells were stained for viability with Live/Dead Fixable Aqua (Invitrogen) in a final volume of 50 μL/well. Cells were incubated for 20 minutes at room temperature in the dark. After the incubation period ended, 150 μL PBS was added to the wells, and the plate was centrifuged for 5 minutes at 450 g. The supernatant was discarded, and each well was stained with anti-CD45-PE/CF594 in a final volume of 50 μL/well. Cells were incubated for 15 minutes at 4 °C in the dark. After the incubation, 150 μL PBS was added to each well, and the plate was centrifuged for 5 minutes at 450 g. The supernatant was discarded. Cells were resuspended in 200 μL of PBS and transferred into FACS tubes. The FACS analysis was conducted with a Celesta cytometer (BD Biosciences). Since the viability test of Aqua LIVE/DEAD Staining was used to differentiate the dead cells, the negative population was selected to continue the analysis. CFSE staining to identify tumour cells and CD45 staining to identify the PBMCs were gated. The tumour cell killing ratio was obtained by dividing the SW480-CFSE^+^CD45^-^ population counts following HK*Mtb* training by the counts obtained in the untrained condition; to facilitate comparison between individuals, all killing assay data were normalized to the average killing obtained from untrained HV.

#### 2.2.8 Statistical analysis

To evaluate the data, various statistical analyses were conducted. Primarily, outlier identification was applied and removed from the analysis by the ROUT test. Then, to assess normality, the Shapiro-Wilk test was applied to all populations. For further analysis between HV and CC patients, the unpaired *t*-test or Mann-Whitney test was used according to the data obtained from the Shapiro-Wilk test. The analyses within the HV or CC patients were paired and then analyzed with a paired *t*-test or Wilcoxon test according to the data obtained from the Shapiro-Wilk test. All FACS data were analyzed with FlowJo Software V10.6.2 (BD Life Sciences) and graphed via Prism GraphPad V8.0.1 (GraphPad Software, Inc.).

## 3 Results

### 3.1 PBMCs from colon cancer patients trained with HK*Mtb* exhibit heterologous secondary inflammatory responses

**Figure 1:**
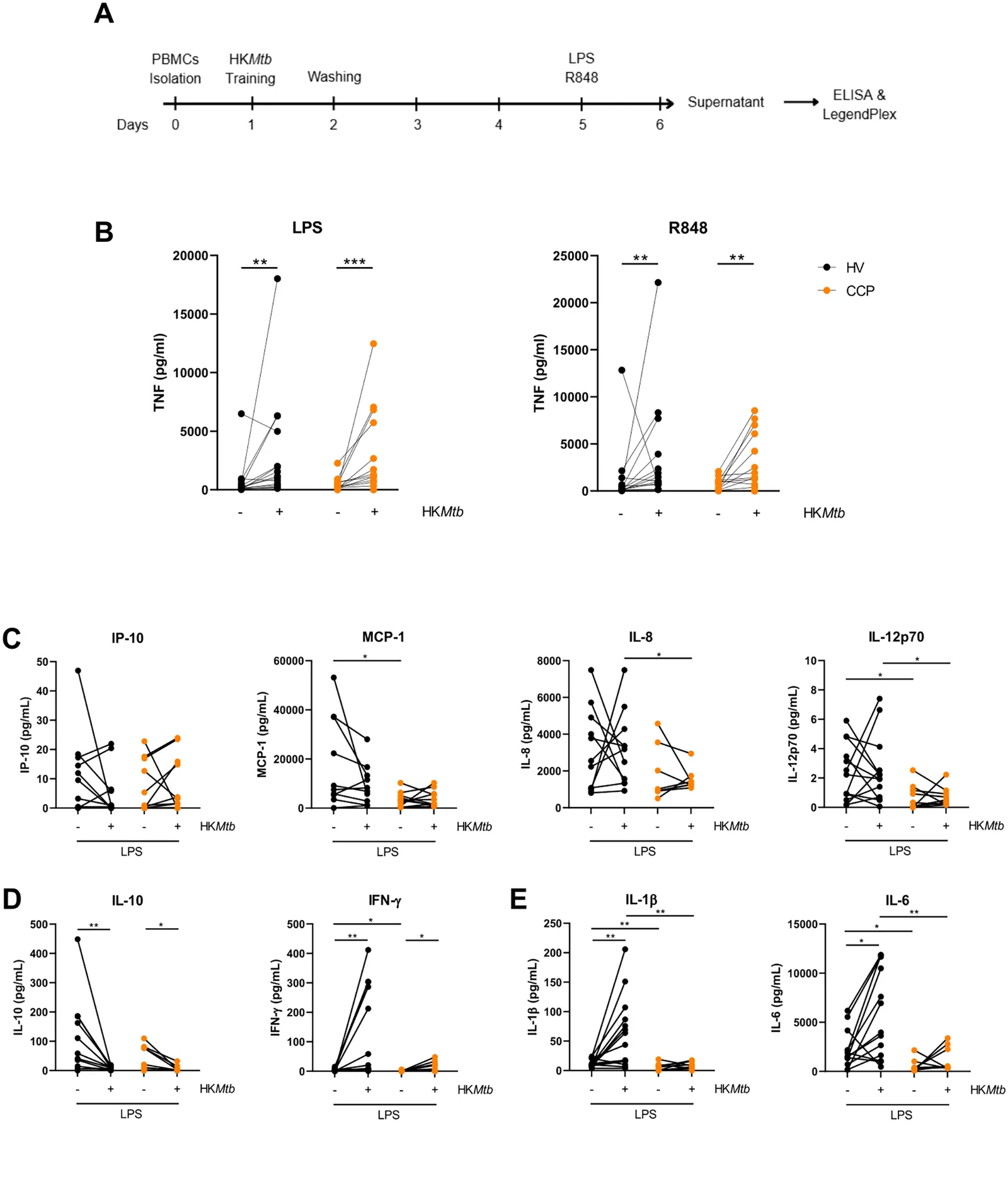
PBMCs from colon cancer patients trained with HK*Mtb* exhibit heterologous secondary inflammatory responses. **(A)** Experimental design of *in vitro* HK*Mtb* training model. **(B)** PBMCs were trained with 10 μg/mL HK*Mtb* for 1 day, washed, and rested for 3 days before LPS (left panel) and R848 (right panel) stimulation on the remaining adherent PBMCs for one more day; supernatants were collected and analysed for TNF by enzyme-linked immunosorbent assay (ELISA). **(C–E)** PBMCs were trained with 10 μg/mL HK*Mtb* for 1 day, washed, and rested for 3 days before LPS stimulation for one more day. ELISA or a multiparametric cytokine determination kit (LegendPlex) was used to analyse cytokine production. Each dot represents a different individual subject, HV in black and CC patients in orange. Untrained and HK*Mtb*-trained conditions were compared using either a paired *t*-test or a Wilcoxon test; meanwhile, the comparison between HV and CC patients was analysed with an unpaired t-test or Mann-Whitney test, depending on the normality of distribution as determined by the Shapiro-Wilk test. (∗*p* < 0.05; ∗∗*p* < 0.01; ∗∗∗*p* < 0.001).

In our previous study, we demonstrated that HK*Mtb* induced TI in human PBMCs and purified monocytes from healthy volunteers (HV) *in vitro*, as well as in preclinical mouse models *in vivo* (33). Aiming to address the translation relevance of these findings in cancer, we evaluated HK*Mtb*-induced training in PBMCs from a cohort of colon cancer patients (CC patients) (**Table 1**). PBMCs were obtained from fresh blood samples from these individuals, which were then exposed to HK*Mtb*. After one day of incubation, non-adherent cells were washed off, and attached cells were rested for 3 days before secondary stimulation (**Figure 1A**).

To assess the response to HK*Mtb-*induced training, cells were secondarily stimulated with LPS or R848, and TNF levels were first measured in the supernatants. HK*Mtb*-trained adherent PBMCs from both HV and CC patients showed increased TNF production in response to both heterologous stimuli (**Figure 1B**). These results indicate that PBMCs from CC patients retain the capacity to undergo HK*Mtb*-induced training.

We next extended this analysis by evaluating a broader cytokine panel to further characterise the trained immune response. Supernatants collected after secondary LPS stimulation showed that MCP-1, IP-10, IL-8, and IL-12p70 were not significantly affected by HK*Mtb* training (**Figure 1C**). However, CC patients and HV displayed differences in some of these cytokines, including MCP-1 under untrained conditions, IL-8 after HK*Mtb* stimulation, and IL-12p70 across both untrained and trained conditions (**Figure 1C**).

By contrast, a training effect was observed for IL-10 and IFN-γ in both HV and CC patients, although a slight baseline difference in IFN-γ was detected between untrained groups (**Figure 1D**). Notably, IL-1β and IL-6 displayed markedly different patterns between HV and CC patients. Both cytokines showed lower baseline levels in CC patients than in HV (**Figure 1E**). Importantly, HK*Mtb* pre-treatment enhanced IL-1β and IL-6 production only in HV, whereas no training effect was observed in PBMCs from CC patients (**Figure 1E**).

Overall, these results indicate that PBMCs from CC patients retain the ability to develop HK*Mtb*-induced TI, as evidenced by enhanced TNF production after heterologous stimulation. However, this response is not fully equivalent to that observed in HV, as CC patients display a distinct cytokine profile characterized by altered baseline levels and selective impairment in the amplification of key pro-inflammatory cytokines, including IL-1β and IL-6. Together, these findings suggest that while TI can be induced in the context of colon cancer, the trained response in CC patients is functionally reshaped, likely reflecting disease-associated immune dysregulation.

### 3.2 HK*Mtb*-induced training enhances *in vitro* tumour-killing capacity on both HV and CC patients

**Figure 2:**
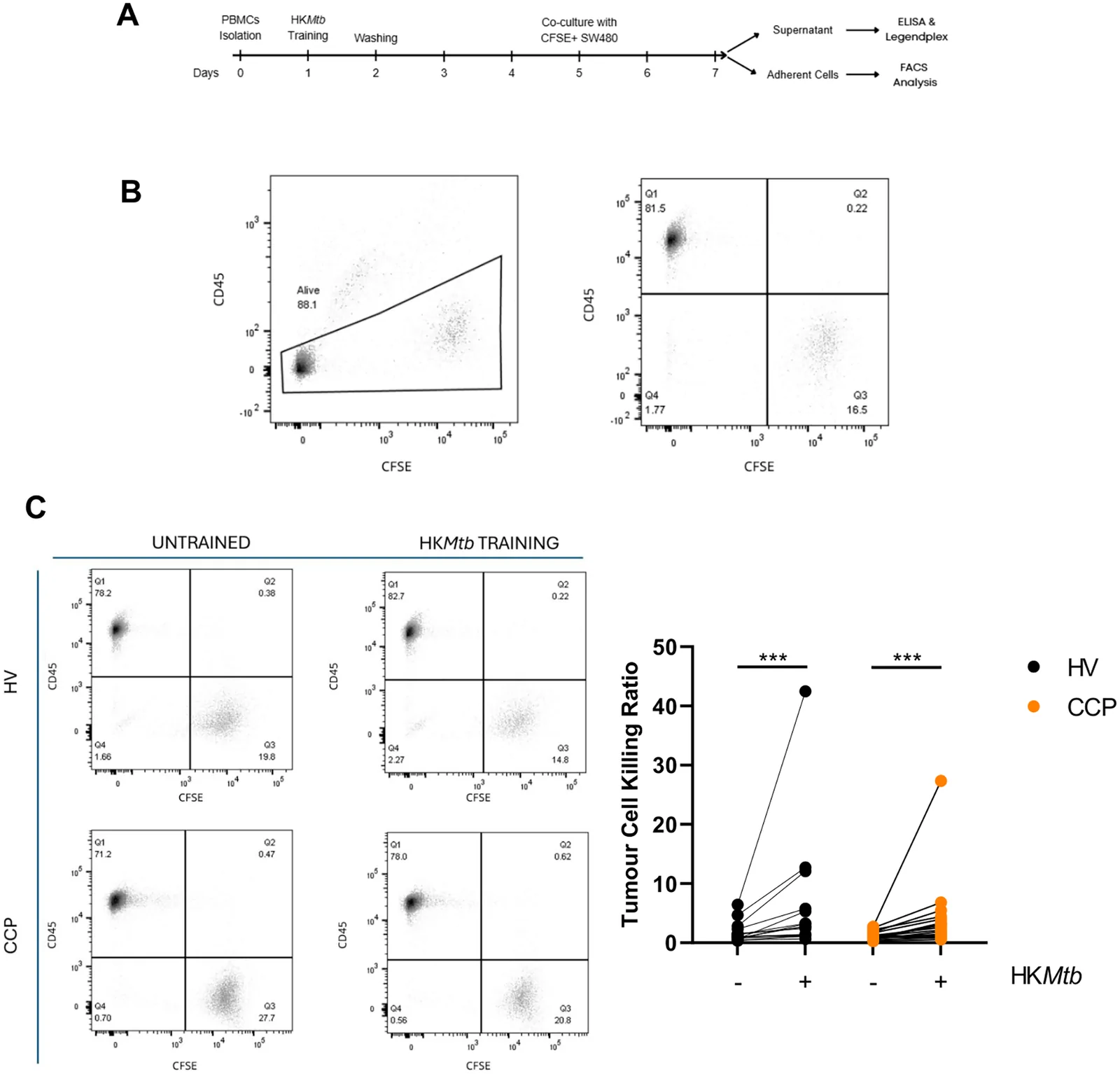
Training with HK*Mtb* enhances tumour-killing capacity in both healthy volunteers and colon cancer patients. **(A)** Experimental design of *in vitro* killing assay. PBMCs trained with HK*Mtb* or untrained were co-cultured with CFSE+ SW480 colon cancer cell line for 2 days. Cell count and composition were analyzed by flow cytometry (FACS). **(B)** FACS gating strategy. Live/Dead Fixable Aqua Dead Cell Stain Kit was used to select live cells (left panel). Subsequently, CFSE+ cells were gated to identify SW480 cells, and CD45+ cells for PBMCs (right panel). **(C)** After the gating was applied to both HV and CC patients, under untrained and trained conditions, the CFSE+CD45- population (Q3) was analyzed to determine the killing activity. A representative example is shown (left panel). The tumour cell killing ratio was calculated based on the remaining SW480 cells in each group (right panel), normalized to the average killing obtained from untrained HV. Each dot represents a different individual subject. Untrained and HK*Mtb*-trained conditions were compared using a paired t-test; meanwhile, the comparison between CC patients and HV was analysed with an unpaired t-test. (∗∗∗*p* < 0.001).

In line with previous evidence showing that TI inducers may have therapeutic potential in selected cancer settings (16, 17), we assessed the effect of HK*Mtb*-induced training on the PBMCs’ tumour-killing capacity against colon cancer cells. To this end, we established an *in vitro* killing assay based on a co-culture of PBMCs previously exposed to HK*Mtb* and the CFSE-labelled human colon cancer cell line SW480 (**Figure 2A**). After 2 days of co-culture, the adherent cellular fraction was characterized by flow cytometry to quantify remaining live tumour cells and adherent PBMCs, while the cytokine profile was analyzed in culture supernatants. Tumor-killing capacity was quantified as the difference between the number of viable SW480 cells (CFSE^+^CD45^-^) between untrained and HK*Mtb*-trained conditions (**Figure 2B**). This assay demonstrated that the tumour-killing capacity of both HV and CC patients improved when PBMCs were trained with HK*Mtb* 3 days before the co-culture (**Figure 2C**).

**Figure 3:**
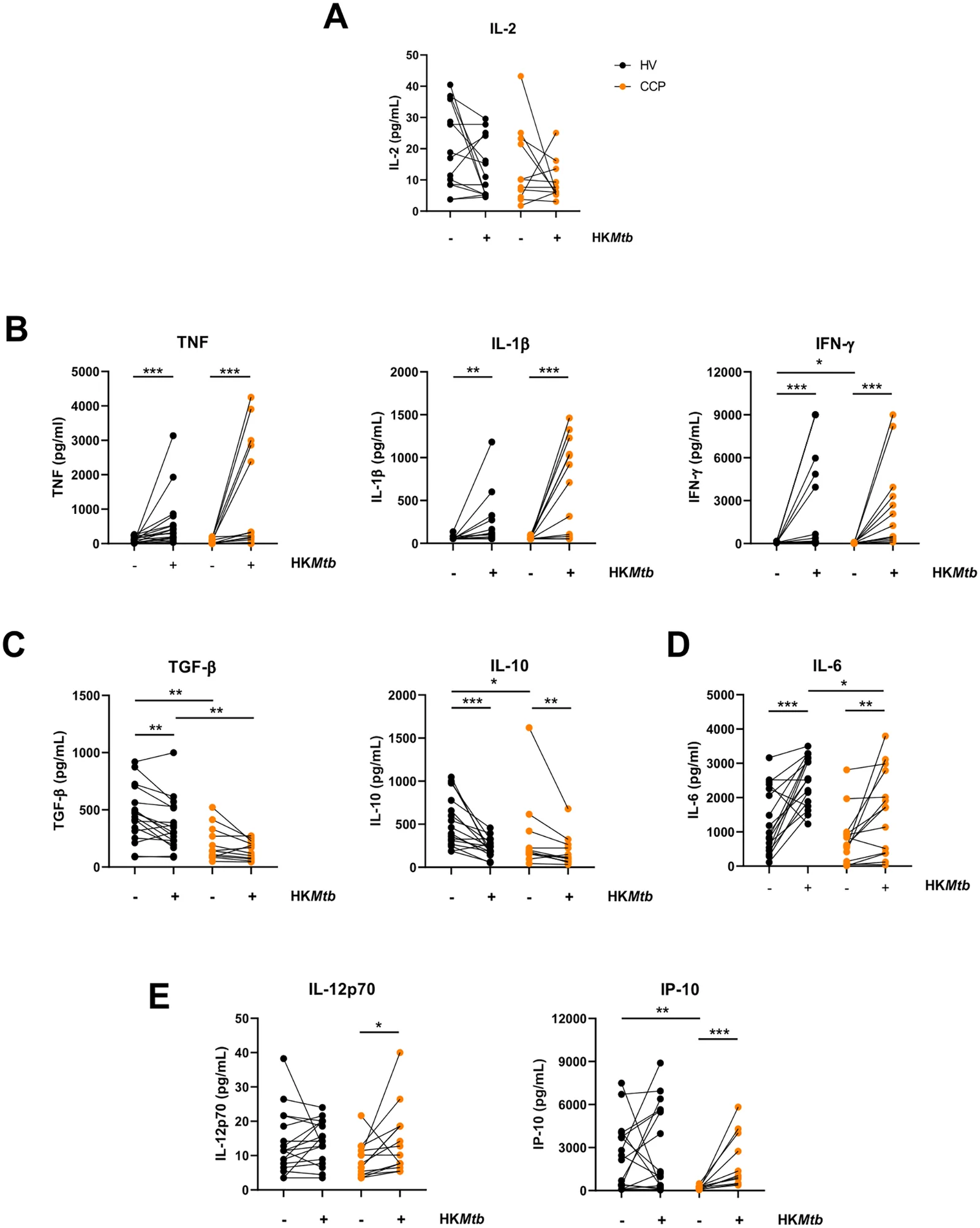
Cytokine profile following *in vitro* killing assay with HK*Mtb*-trained PBMCs. Supernatants collected after the killing assay described in **Figure 2** were analysed. Enzyme-linked immunosorbent assay (ELISA) (TNF, IL-6) and multiparametric cytokine determination kit (LegendPlex) were used and plotted as **(A)** IL-2, **(B)** TNF, IL-1β, IFN-γ, **(C)** TGF-β, IL-10, **(D)** IL-6, and **(E)** IL-12p70 and IP-10. Each dot represents a different individual subject. Untrained and HK*Mtb*-trained conditions were compared using either a paired *t*-test or a Wilcoxon test, while the comparison between HV and CC patients was analysed with an unpaired *t*-test or Mann-Whitney test depending on the normality of distribution as determined by the Shapiro-Wilk test. (∗*p* < 0.05; ∗∗*p* < 0.01; ∗∗∗*p* < 0.001).

The analysis of supernatants collected after the killing assay showed that IL-2 levels were not affected by training in either group (**Figure 3A**). However, proinflammatory cytokines, including TNF, IL-1β, and IFN-γ, displayed a robust trained response in both HV and CC patients after HK*Mtb* training (**Figure 3B**), although baseline IFN-γ levels were again slightly lower in CC patients, consistent with the pattern observed before LPS stimulation.

HK*Mtb* training reduced TGF-β levels in HV, while PBMCs from CC patients showed significantly lower TGF-β levels than HV, regardless of HK*Mtb* pre-stimulation, with no additional training effect for this cytokine (**Figure 3C**). IL-10 was also reduced following HK*Mtb* training in both HV and CC patients, with CC patients showing lower basal expression under untrained conditions (**Figure 3C**). Furthermore, HK*Mtb*-induced training elicited a comparable relative increase in IL-6 production in both HV and CC patients. However, the absolute levels of IL-6 produced after training were significantly higher in PBMCs from HV than in those from CC patients (**Figure 3D**).

Finally, HK*Mtb*-induced training specifically enhanced IL-12p70 and IP-10 production in CC patients. These mediators were not affected by HK*Mtb* pre-stimulation in PBMCs from HV (**Figure 3E**). In contrast, PBMCs from CC patients showed increased IL-12p70 and IP-10 after training. Notably, although IP-10 levels were basally lower in CC patients, this difference was overcome by HK*Mtb* training (**Figure 3E**).

Of note, cytokines were basically not detected in supernatants from SW480 tumor cells cultured alone (data not shown), indicating that the cytokine responses analyzed come from PBMCs following their interaction with the tumor cells.

These results demonstrate that HK*Mtb*-induced training enhances the tumour-killing capacity of PBMCs from both HV and CC patients, indicating that TI can functionally boost antitumour responses in the colon cancer setting. This effect is associated with a reinforced pro-inflammatory cytokine profile, including increased TNF, IL-1β, and IFN-γ production, together with reduced immunoregulatory mediators such as IL-10 and TGF-β. Notably, PBMCs from CC patients exhibit a distinct response pattern, characterized by selective induction of IL-12p70 and IP-10 following training, suggesting a partial rewiring of trained responses in the context of colon cancer.

### 3.3 HK*Mtb*-induced training reverses the *in vitro* lack of response to anti-PD1 in CC patient-derived PBMCs

**Figure 4:**
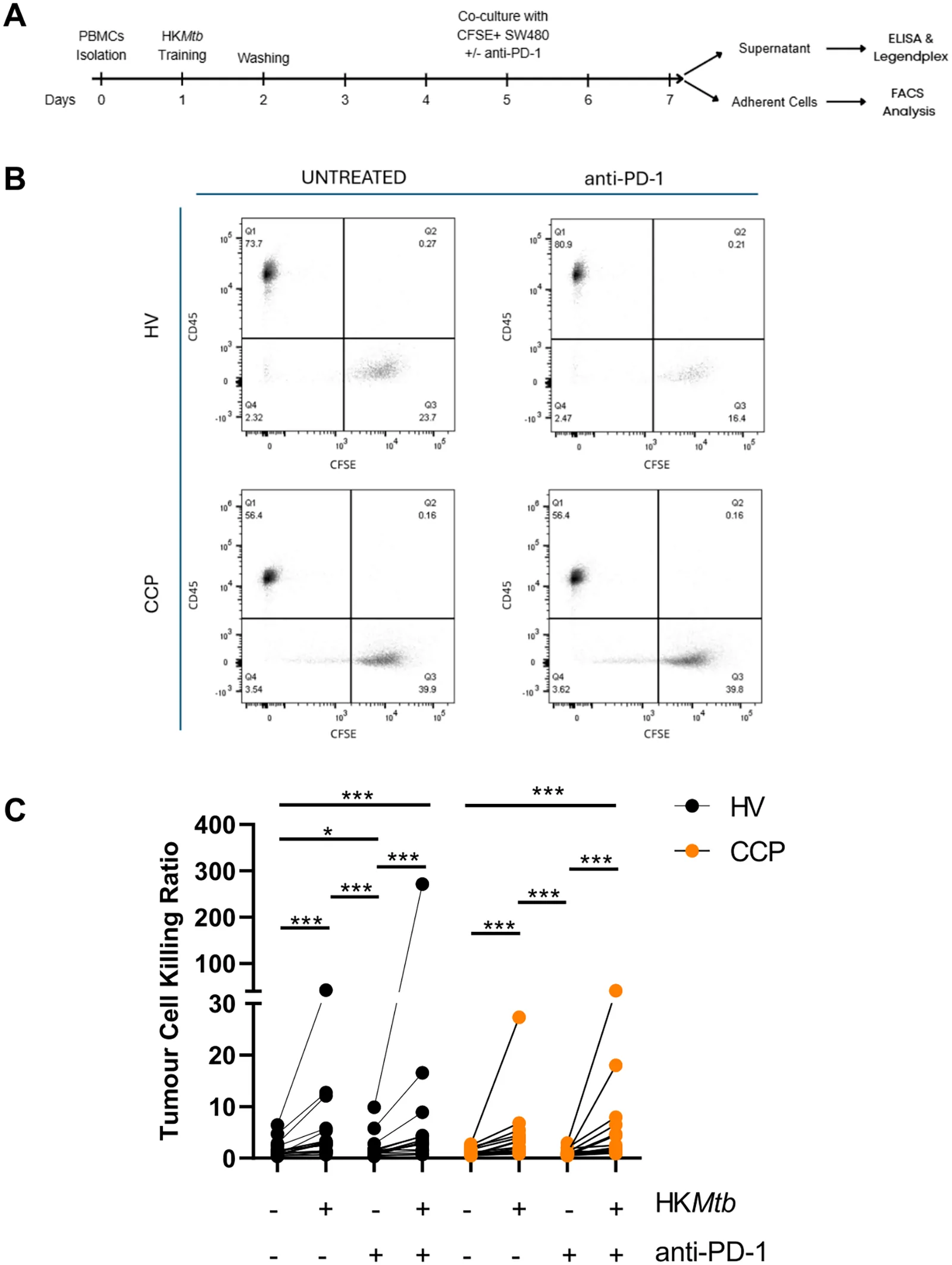
HK*Mtb*-induced training reverted the lack of *in vitro* response to anti-PD1 in PBMCs from colon cancer patients. **(A)** Experimental design of *in vitro* killing assay in the presence or absence of anti-PD-1 immunotherapy. PBMCs trained with HK*Mtb* or untrained were co-cultured with the CFSE+ SW480 colon cancer cell line for 2 days in the presence or absence of anti-PD-1. Cell count and composition were analyzed by flow cytometry (FACS). **(B)** After gating was applied to both HV and CC patients, in the absence and presence of anti-PD-1, the CFSE+CD45- population (Q3) was analyzed to determine the killing activity. **(C)** The tumour cell killing ratio was calculated considering the remaining SW480 cells in each group, normalized to the average killing obtained from untrained HV. Each dot represents a different individual subject. All experimental conditions within HV and CC patients (-/+ HK*Mtb*, -/+ anti-PD-1) were compared using a paired *t*-test or a Wilcoxon test; meanwhile, the comparison between HV and CC patients was analysed with an unpaired *t*-test or Mann-Whitney test, depending on the normality of distribution as determined by the Shapiro-Wilk test. (∗*p* < 0.05; ∗∗*p* < 0.01; ∗∗∗*p* < 0.001).

Having established that HK*Mtb* training improves the *in vitro* tumour-killing capacity of PBMCs from both HV and CC patients, we next examined whether HK*Mtb*-induced training could be combined with anti-PD-1 immunotherapy using Nivolumab. The killing assay was therefore performed in the presence or absence of anti-PD-1 antibody (**Figure 4A**). Anti-PD-1 treatment enhanced the tumour-killing capacity of PBMCs from HV, validating this assay as a platform to evaluate the antitumour effect of checkpoint blockade (**Figures 4B, C**). Importantly, PBMCs from CC patients did not show such a comparable tumour-killing response to anti-PD-1 treatment. In line with these results, all CC patients were proficient in the mismatch repair system (pMMR) and showed microsatellite stability (MSS). Nevertheless, HK*Mtb* training enhanced the tumour-killing capacity of PBMCs derived from CC patients, regardless of the presence of anti-PD-1. Indeed, *HKMtb*-trained PBMCs from both HV and CC patients showed stronger killing compared to anti-PD-1 alone (**Figure 4C**). These results indicate that HK*Mtb*-induced training elicits antitumor activity in PBMCs from CC patients that lack *in vitro* responses to anti-PD-1.

**Figure 5:**
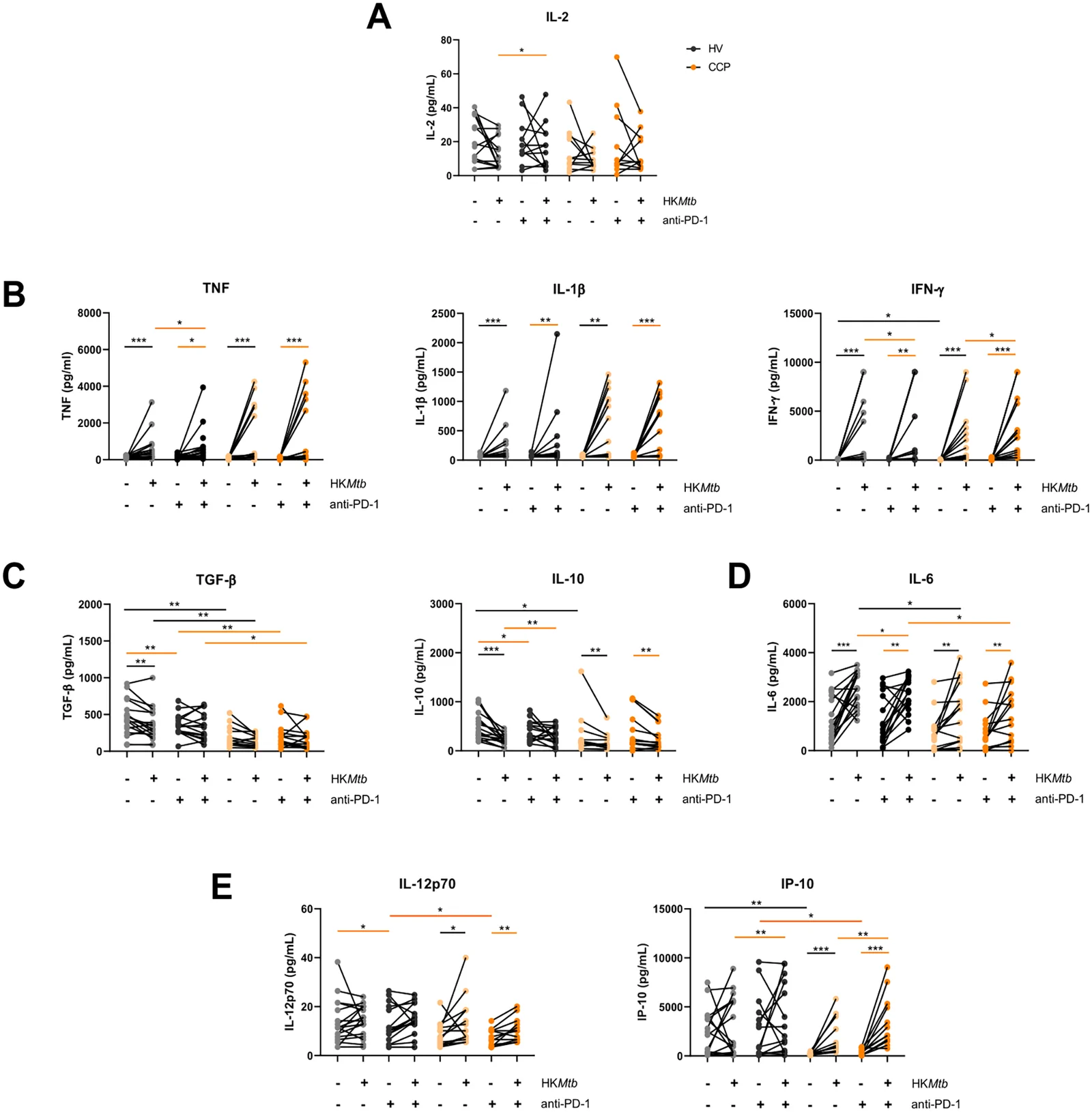
Cytokine profile following *in vitro* killing assay with both HK*Mtb* training and anti-PD-1 treatment. Supernatants collected after the killing assay described in **Figure 4** were analysed. Cytokines were quantified using ELISA (TNF, IL-6) or a multiparametric cytokine determination kit (LegendPlex) and are shown as follows: **(A)** IL-2, **(B)** TNF, IL-1β, IFN-γ, **(C)** TGF-β, IL-10, **(D)** IL-6, and **(E)** IL-12p70 and IP-10. Each dot represents an individual subject. Untrained and HK*Mtb*-trained conditions were compared using either a paired *t*-test or a Wilcoxon test; meanwhile, the comparison between HV and CC patients was analysed with an unpaired *t*-test or a Mann-Whitney test, depending on the normality of distribution as determined by the Shapiro-Wilk test. Statistical differences related to anti-PD-1 treatment are highlighted in orange (∗*p* < 0.05; ∗∗*p* < 0.01; ∗∗∗*p* < 0.001).

Given the differences observed in tumour-killing capacity between HV and CC patients in the presence of anti-PD-1, we analysed supernatants from these killing assays to assess potential differences in cytokine production. For clarity, statistical differences related to anti-PD-1 treatment are highlighted in orange (**Figure 5**). Although IL-2 production was not altered by training alone, this cytokine showed a specific pattern in HV when HK*Mtb* training was combined with anti-PD-1 (**Figure 5A**). Pro-inflammatory cytokines induced by HK*Mtb* training in both HV and CC patients, including TNF, IL-1β, and IFN-γ, were not induced by anti-PD-1 treatment alone. However, a synergistic effect with HK*Mtb* was observed for TNF in HV and IFN-γ in both groups (**Figure 5B**).

The production of the regulatory cytokines TGF-β and IL-10 was reduced following anti-PD-1 treatment, specifically in HV, with no major effect in CC patients beyond HK*Mtb* pre-treatment for IL-10 (**Figure 5C**). A further HV-specific effect was observed for IL-6, which was reduced after combined HK*Mtb*-induced training and anti-PD-1 treatment, whereas anti-PD-1 alone had no effect in either group (**Figure 5D**).

Distinct patterns were also observed for IL-12p70 and IP-10 production. These cytokines showed lower levels in CC patients than HV after anti-PD-1 treatment alone (**Figure 5E**). Nevertheless, IL-12p70 and IP-10 production increased specifically in PBMCs from CC patients following HK*Mtb*-induced training, either independently of anti-PD-1 treatment for IL-12p70 or further boosted in combination with anti-PD-1 for IP-10 (**Figure 5E**).

Altogether, these results demonstrate that HK*Mtb*-induced TI functionally reverses the *in vitro* lack of response to anti–PD-1 of PBMCs from CC patients, restoring their tumour-killing capacity. While anti–PD-1 alone was ineffective in CC patients, HK*Mtb* training consistently promoted robust antitumour activity, independent of anti–PD-1 checkpoint blockade. This functional improvement was accompanied by a distinct cytokine remodeling, including selective induction of IL-12p70 and IP-10 in CC patients, suggesting a partial reprogramming of immune responses in the colon cancer setting.

## 4 Discussion

Trained immunity is a functional reprogramming driven by epigenetic mechanisms and metabolic rewiring, which primes innate immune cells to mount enhanced proinflammatory responses upon secondary heterologous challenges (41). The protective effect of TI against diverse infections has highlighted its potential to combat pathogens for which no specific vaccines are available, or to enhance protection when combined with conventional vaccines, giving rise to the concept of TI-based vaccination (42). BCG is probably the most studied TI-inducer in clinical settings. However, BCG carries an inherent risk of disseminated infection in immunocompromised patients, which restricts its broader application (18, 19). In response to these safety concerns, gamma-irradiated BCG (43) or alternative heat-killed vaccines have been explored as potential TI inducers (27). Nevertheless, inactivated BCG shows some limitations in inducing trained responses. Despite irradiated BCG inducing TI *in vitro* in human monocytes, coupled with some key molecular training features, these effects were weaker than those induced by live BCG, with HK-BCG vaccination in volunteers having only minimal effects on innate immunity (43). Furthermore, experimental work comparing live and HK-BCG reported that live BCG, but not HK-BCG, induced TI in macrophages and heterologous protection in a murine model of *E. coli* sepsis (44), suggesting that bacterial viability may contribute to optimal induction of TI by BCG. Aiming to overcome these limitations, we showed in our recent study that heat-killed *Mycobacterium tuberculosis* (HK*Mtb*) induces TI in human PBMCs *in vitro* and in preclinical *in vivo* mouse models (33). Although TI has been studied as a potential antitumour strategy (45), studies in colon cancer remain restricted to preclinical mouse models (20, 40). These considerations position this study as a timely effort to explore TI inducers with translational potential for CC patients.

Given the broad unresponsiveness of colon cancer to immunotherapy, we focused on pMMR/MSS CC patients, who are less susceptible to benefit from immune checkpoint blockade (46). In this context, we investigated whether PBMCs from CC patients could be trained by HK*Mtb*, as previously observed in HV (33). PBMCs from both groups were exposed to HK*Mtb* and, after a resting period, adherent cells were stimulated with LPS or R848 as secondary heterologous challenges. TNF was used as a main readout of TI, as enhanced TNF production upon restimulation reflects epigenetic priming of pro-inflammatory gene promoters, including H3K4me3 enrichment, and is therefore indicative of functional rewiring rather than transient activation (15). TNF levels increased in both HV and CC patients in response to both stimuli, indicating that PBMCs from CC patients retain training capacity comparable to that of HV. Of note, we already demonstrated that HK*Mtb*-induced training recapitulated key metabolic and epigenetic features of the trained process, such as lactate accumulation and HIF-1α dependency, and enrichment of H3K4me3 at the *TNFA* promoter, respectively (33). Altogether, the observed phenotype in CC patients is consistent with TI. This finding is relevant because it establishes proof of concept that immune cells from these patients may be amenable to TI-based approaches.

Because LPS signals through Toll-Like Receptor (TLR)-4, which induces many signaling pathways, we further investigated the levels of other pro- and anti-inflammatory cytokines. IL-1β and IL-6 showed a parallel trend with TNF in HV after training with HK*Mtb* and secondary LPS stimulation, consistent with their induction through the same transcription factors (NF-κB and AP-1) (47). Interestingly, both IL-1β and IL-6, along with MCP-1, IL-12p70, and IFN-γ, showed reduced baseline levels in CC patients, in line with previous data (48). Among these cytokines, HK*Mtb* pretreatment enhanced IFN-γ production, supporting a positive effect of HK*Mtb* training in overcoming these reduced baseline levels in CC patients. Overall, this specific TI profile in CC patients suggests that the trained machinery is not fully preserved in these patients, possibly reflecting disease-associated epigenetic or functional constraints (49).

Interestingly, IL-12p70 showed no significant modulation after training in either group, while its production was lower in CC patients than in HV. This finding is relevant because myeloid-derived IL-12 stimulates NK and T cells, promotes IFN-γ production, and serves as a bridge between innate and adaptive immunity (50). Reduced IL12p70 levels may therefore contribute to the overall lower baseline cytokine production observed in CC patients, particularly for IL-12-dependent mediators such as IFN-γ. In line with this interpretation, recent studies indicate that IL-12 signaling initiates early IFN-γ production after activation, primarily by NK cells, while sustained IFN-γ responses at later time points are predominantly driven by activated T cells, indicating that IL-12 functions as an early instructive signal rather than a continuously required stimulus (51, 52). Nevertheless, IP-10 (CXCL-10), a canonical IFN-γ–inducible chemokine, was not elevated in parallel with IFN-γ. In this sense, studies showed that when Interferon Response Factor (IRF)-1 stimulation happens at low concentrations and duration, although it induces IFN-γ production, IP-10 needs larger concentrations and longer stimulation to be produced (53). This is consistent with our results, given the 24-hour LPS secondary stimulation of our workflow, and the relatively low IL12p70 production detected, especially compared with the levels detected in the tumour-killing assays.

In contrast to proinflammatory cytokines, IL-10 did not show any baseline differences between HV and CC patients, yet showed a parallel decrease after training with HK*Mtb* in both groups. Recent studies indicate that high IL-10 levels drive tolerance in primary human monocytes and restrain TI at the cellular function and gene expression levels (54). Accordingly, the reduction in IL-10 observed here after training in both HV and CC patients supports the presence of shared features of the TI programme across both groups.

Having established that HK*Mtb* induces specific trained responses in PBMCs from CC patients, we next addressed whether this treatment could enhance their tumour-killing capacity. To this end, we performed an *in vitro* killing assay using a co-culture of PBMCs and the human colon cancer cell line SW480, which in these settings acted as the secondary heterologous challenge. HK*Mtb*-induced training improved the tumour-killing capacity in PBMCs from both HV and CC patients, supporting this approach as an antitumour treatment against colon cancer.

Cytokine profiling of the supernatants further supported the induction of training, showing a shared upregulation of TNF, IL-1β, IFN-γ, and IL-6, together with downregulation of IL-10 in both groups. In addition, baseline differences between HV and CC patients in IL-10 and IP-10 were balanced after HK*Mtb* training, with a specific boost in IP-10 production in CC patients. By contrast, baseline differences observed in TGF-β between groups persisted after training, with no detectable effect of HK*Mtb* in CC patients. Increased IL-12p70 and IP-10 levels following HK*Mtb*-induced training in CC patients may be linked to reduced TGF-β levels, as IL-12p70 induces IP-10 and both have been associated with inhibition of TGF-β expression through Smad7 induction (55, 56). Overall, these cytokine expression patterns support the induction of an active antitumour microenvironment following HK*Mtb*-induced training, which associates with enhanced tumour-killing capacity.

Of note, in the tumour-killing assays, we observed different IL-12p70 and IFN-γ expression patterns compared with the training protocol using LPS secondary stimulation. A key difference between assays is the time of analysis: 24 hours for LPS and 48 hours for the killing. As mentioned before, the incubation period is crucial for IP-10 and IFN-γ levels. In the initial heterologous assay, the IP-10 levels were not yet affected, and IL-12p70 was already neutralized, with a detectable impact of HK*Mtb* training on IFN-γ. However, in the killing assay supernatants, the levels of IP-10 were boosted after training in parallel with IFN-γ, particularly in CC patients, illustrating the relevance between the timing of the analysis and the cytokine levels.

Considering our data pointing to an immunotherapeutic effect of HK*Mtb*-induced training, and that CC patients are unresponsive to immune checkpoint blockade-based immunotherapy, we investigated the effects of anti-PD-1 in combination with HK*Mtb*. PBMCs from HV exhibited increased killing capacity when exposed to anti-PD-1; strikingly, the tumour-killing capacity of PBMCs from CC patients was unaffected by this immunotherapy. Nonetheless, HK*Mtb*-induced training boosted the tumour-killing capacity in both groups regardless of the presence of anti-PD-1, even reaching higher tumouricidal activity than with this blocking antibody. These results recapitulate clinical evidence, considering that MSS-CC patients are unresponsive to anti-PD-1 (46). Furthermore, these results indicate that HK*Mtb*-induced training may revert the *in vitro* lack of response to anti-PD-1 in CC patients, as evidenced by enhanced tumour cell killing.

Further analyses of the supernatants to evaluate the cytokine profile during the killing assay showed that in HV, anti-PD-1 treatment alone had a specific impact on TGF-β, IL-10, and IL-12p70, while none of these behaviours were detected in CC patients. The reduced IL-12p70 and IP-10 expression may contribute to the lack of anti-PD-1 response in CC patients, but additional mechanistic studies (*e.g.*, neutralization of these cytokines) are needed to confirm this hypothesis.

In line with the results obtained in the tumour-killing assay, the HK*Mtb*-induced training effect prevailed over anti-PD-1 immunotherapy regarding proinflammatory cytokine production. Nevertheless, specifically in HV, reduced expression of the regulatory cytokines TGF-β and IL-10, coupled with increased IL-2 and TNF, was observed following the combined treatment. This synergistic effect was shared by both HV and CC patients for IFN-γ and IP-10. Nonetheless, the combination of HK*Mtb* and anti-PD-1 specifically enhanced the production of IL-12p70 and IP-10 in CC patients. Altogether, these results illustrate that training with HK*Mtb* recovered several baseline differences observed in CC patients during the tumour-killing assay, releasing some limitations observed in response to anti-PD-1 treatment. Of note, the pro-inflammatory profile observed in CC patients in terms of increased IL12-p70 and IP-10 is of particular relevance, as this phenotype has been linked to highly immunogenic and potent anti-tumour responses (57).

While this study establishes strong proof of concept, we must acknowledge that cytokine measurements were performed on total supernatants derived from co-cultures of PBMCs and SW480. Although we confirmed that these cytokines were not produced by SW480 tumor cells themselves, future studies using flow cytometry will allow us to dissect which PBMC subpopulations are responsible for the production of different cytokines. Furthermore, the limited sample size and the exploratory nature of the cohort preclude definitive conclusions regarding patient stratification or clinical responsiveness to PD-1 blockade.

Nevertheless, to our knowledge, this is the first report showing that PBMCs from CC patients can develop TI linked to increased tumour-killing capacity. In addition, the lack of *in vitro* response to immune checkpoint inhibition observed in PBMCs from CC patients was reverted by TI induction. Collectively, these findings highlight the potential of HK*Mtb* as a clinically relevant TI inducer in colon cancer. Nonetheless, *in vivo* studies will be required to establish the translational relevance of these findings to clinical resistance to PD-1 blockade.

## 5 Conclusion

A substantial proportion of CC patients remain refractory to current immunotherapies, underscoring the need for novel therapeutic strategies. Although the broader oncology field has begun to recognise the impact of TI, its application in colon cancer has been limited by safety concerns related to classical TI inducers and by the lack of clinical validation. This work provides proof of concept that HK*Mtb*-induced TI enhances the tumour-killing capacity of CC patient-derived PBMCs. Importantly, this approach reverted the lack of anti-PD-1 responsiveness *in vitro*, highlighting the potential of targeting innate immune memory as a complementary strategy against immunotherapy resistance in colon cancer. Collectively, these findings support the concept that TI can bypass unresponsiveness to checkpoint inhibition, providing a proof of concept for a complementary immunotherapeutic strategy in CC patients.

## Data Availability

The raw data supporting the conclusions of this article will be made available by the authors, without undue reservation.
