# Supplementary material for: Heat-killed Mycobacterium tuberculosis-induced trained immunity elicits antitumor activity in PBMCs from colon cancer patients with unresponsiveness to anti-PD-1 in vitro
Source: Front Immunol. 2026 Sep 11;17:1911032. doi: 10.3389/fimmu.2026.1911032 (PMC13612437; doi:10.3389/fimmu.2026.1911032)
Supplement: Supplementary file 1 [file SupplementaryFile1.docx]

**Supplementary Material**

**Table S1:** List of antibodies

| **Detection** | **Fluorescence** | **Company** | **Catalog Number** |
| --- | --- | --- | --- |
| CD45 | PE-CF594 | BD Horizon | #562279 |
| CFSE | BB-515 | Invitrogen | #C34554 |

**Table S2:** List of equipment

| **Material** | **Company** | **Catalog Number** |
| --- | --- | --- |
| 6-well Treated Surface Culture Plate | SPL Life Sciences | #30006 |
| 96-well Treated Surface Flat Bottom Plate | Avantor | #734-2327 |
| 96-well Non-treated Surface Round Bottom Plate | Avantor | #734-2328 |
| T75 cell culture flask | Corning | # CLS430641U |
| 50 mL tube | Deltalab | #429027 |
| 25 mL tube | Deltalab | #309222 |
| 1,5 mL tube | Deltalab | #200400P |
| Electric Pipette Stripettor™ Ultra Pipet | Corning | #4099 |
| Pipette (1000μl, 200μl, 20μl, 10μl) | Avantor | #613-7141 |

**Table S3:** List of chemicals and reagents

| **Chemical or Reagent** | **Company** | **Catalog Number** |
| --- | --- | --- |
| Ficoll-Plus | GE Healthcare | # 17-1440-03 |
| Phosphate-Buffered Saline (PBS) | Sigma-Aldrich | # P4417-100TAB |
| Fetal Bovine Serum (FBS) | Thermo Fisher Scientific | # 11560636 |
| Trypan-Blue | Gibco | # 15250061 |
| RPMI 1640 Medium | Thermo Fisher Scientific | # 11594506 |
| Penicillin/Streptomycin | Gibco | # 15140-148 |
| Heat-Killed *Mycobacterium tuberculosis* (HK*Mtb*) | InvivoGen | # tlrl-hkmt |
| L-Glutamine/GlutaMAX | Gibco | # 35050061 |
| Sodium Pyruvate | Gibco | # 11360070 |
| Trypsin/EDTA | Gibco | # 25200056 |
| Lipopolysaccharides (LPS) *Escherichia coli* | Sigma-Aldrich | # L4391 |
| Resiquimod (R848) | InvivoGen | # tlrl-r848 |
| Pharm/Lyse Lysis Buffer | BD Biosciences | # 555899 |
| TrypLE™ Express Enzyme (1X) - No Phenol Red | Gibco | # 12604021 |

**Table S4:** List of commercial assays

| **Assay** | **Company** | **Catalog Number** |
| --- | --- | --- |
| Live/Dead Fixable Aqua Dead Cell Stain Kit | Invitrogen | #L34957 |
| Human IL-6 DUO Set | R&D Systems | # DY206 |
| Human TNF-α DUO Set | R&D Systems | # DY210 |
| LEGENDplex™ Human Essential Immune Response Panel | BioLegend | #740930 |

**Table S5:** List of software

| **Software** | **Company** |
| --- | --- |
| FlowJo Software | BD Life Sciences |
| GraphPad Prism | GraphPad Software, Inc. |
| FACSDiva™ Software | BD Life Sciences |
| Epoch 2 Microplate Spectrophotometer | BioTech |

**Figure S1: Comparable heterologous trained responses after three or five days of HK*Mtb* stimulation**. PBMCs were trained with 10 μg/mL HK*Mtb* for 1 day, washed, and rested for 3 (black dots) or 5 days (blue dots) before LPS stimulation on the remaining adherent PBMCs for one more day; supernatants were collected and analysed for TNF by enzyme-linked immunosorbent assay (ELISA). Each dot represents an individual subject. Based on the normality of distribution as determined by the Shapiro-Wilk test, the Kruskal-Wallis test was applied. (ns, not significant; ∗∗*p* < 0.01).
